# Cigarette smoke-induced oxidative stress activates NRF2 to mediate fibronectin disorganization in vascular formation

**DOI:** 10.1098/rsob.210310

**Published:** 2022-04-27

**Authors:** Jinjiang Xue, Qiong Liao, Man Luo, Chenfeng Hua, Junwei Zhao, Gangfeng Yu, Xiangyu Chen, Xueru Li, Xinchun Zhang, Ruiguo Ran, Fanghui Lu, Yingxiong Wang, Liangjun Qiao

**Affiliations:** ^1^ College of Basic Medicine, Chongqing Medical University, 1 Yixueyuan Road, Chongqing 400016, People's Republic of China; ^2^ Institute of Life Sciences, Chongqing Medical University, 1 Yixueyuan Road, Chongqing 400016, People's Republic of China; ^3^ Key Laboratory of Tobacco Chemistry, Zhengzhou Tobacco Research Institute of CNTC, 2 Fengyang Street, Zhengzhou 450001, People's Republic of China

**Keywords:** cigarette smoke, oxidative stress, NRF2, STAT3, fibronectin, vascular formation

## Abstract

Cigarette smoke significantly induces oxidative stress, resulting in cardiovascular disease. NRF2, a well-known antioxidative stress response factor, is generally considered to play protective roles in cardiovascular dysfunction triggered by oxidative stress. Interestingly, recent studies reported adverse effects of NRF2 on the cardiovascular system. These unfavourable pathogenic effects of NRF2 need to be further investigated. Our work shows that cigarette smoke extract (CSE)-induced oxidative stress disturbs fibronectin (FN) assembly during angiogenesis. Furthermore, this effect largely depends on hyperactive NRF2-STAT3 signalling, which consequently promotes abnormal FN deposition. Consistently, disruption of this pathway by inhibiting NRF2 or STAT3 prevents CSE-induced FN disorganization and vasculature disruption in human umbilical vein endothelial cells or zebrafish. Taken together, these findings demonstrate the cardiovascular dysfunction caused by CSE from a novel perspective that NRF2-dependent signalling engages in FN disorganization.

## Graphical abstract

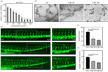

## 1. Introduction

Smoking is a leading cause of cardiovascular diseases such as stroke, atherosclerosis and other diseases [1]. Many of these pathological mechanisms involve the response to oxidative stress because cigarettes are composed of about 6000 components such as NO, CO and particulate matter [2]. For instance, cigarette smoke causes oxidative stress in the initiation of atherosclerosis, which involves endothelial damage, platelet activation, lipid peroxidation and so on [1,3].

An imbalance of oxidative stress, marked by deregulation of reactive oxygen species (ROS), results in diverse vascular pathologies [4,5]. For example, excessive ROS accumulation induces chronic inflammation, extracellular matrix (ECM) disorganization, DNA damage and eventually angiogenesis defects [6]. The downstream effectors of ROS include antioxidant genes such as *NRF2*, *HMOX1, NOS2* and *SOD1* [7,8]. Specifically, *NRF2* has been associated with cardiovascular pathogenesis [8,9], exhibiting protective effects via the induction of a series of antioxidant genes [10,11], but opposite effects were also observed in atherosclerotic exacerbation or myocardial dysfunction [12,13].

NRF2, a member of the Cap'n’Collar transcription factor family, is widely expressed in various tissues at low basal levels and is usually degraded by E3 ubiquitin ligase via binding Kelch-like ECH-associated protein (Keap)1. When cells are under oxidative stress, NRF2 becomes unleashed from Keap1 and translocates into the nucleus to transcriptionally regulate antioxidant responsive element-dependent genes for the maintenance of cellular redox homeostasis [14]. The downstream targets of NRF2 activation also include HOMX1 and NAD(P)H:quinone oxidoreductase-1 (nqo1) [15]. In addition, NRF2 was suggested to regulate tissue-specific fibronectin (FN) expression [16,17], but less is known about its role in FN assembly.

As a major ECM structure protein, the adhesive glycoprotein FN promotes endothelial cell activation, survival, migration and elongation [18–20], which are vital for embryogenesis and vascular development. Mice lacking FN expression exhibit severe cardiovascular defects and show embryo lethality at E8.5 [21]. FN interacts with integrins through the RGD motif (Arg–Gly–Asp) to form fibrils, and the complex further binds with focal adhesion kinase (FAK) or actin to exert various cellular processes [22]. Our previous studies along with other work have revealed that the disruption of FN assembly causes defective pre-chordal plate movements and cardiac infusion in zebrafish via the stat3–efemp2 or snail–integrin pathways [23,24].

Although oxidative stress induced by cigarette smoke mediates multiple vascular dysfunctions, the role of NRF2 in response to cigarette smoke in vascular organization is still not fully understood. It is not known whether NRF2 regulates FN to disturb vascular formation. Here, our data have shown that cigarette smoke extract (CSE) disrupts vasculature formation *in vitro* and *in vivo* through NRF2 signalling. Aberrant NRF2 activation in human umbilical vein endothelial cells (HUVECs) or zebrafish, tightly associated with CSE-stimulated ROS accumulation, leads to disrupted FN assembly via the STAT3 pathway. Consistently, the suppression of NRF2 or STAT3 function by an inhibitor largely restores FN fibrils and vascular formation. In summary, these results provide novel insight into CSE-induced vascular dysfunction that NRF2 serves as the accomplice to FN disorganization.

## 2. Results

### 2.1. Cigarette smoke extract disrupted endothelial organization

**Figure 1.**
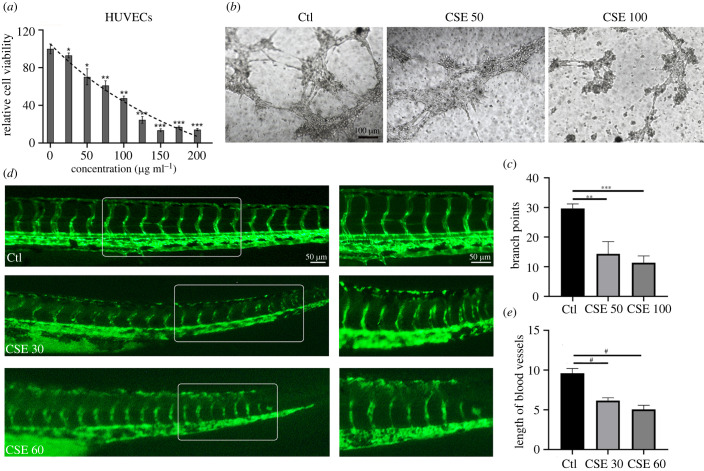
CSE disturbed vasculature formation in HUVECs and zebrafish. (*a*) HUVEC viability after exposure to CSE at different concentrations for 24 h. (*b*) Tube formation assay for HUVECs cultured on Matrigel with CSE treatment. (*c*) Quantification of the vascular branch points formed by HUVECs. (*d*) Representative images of vasculature formation in *flk*::GFP zebrafish treated with CSE for 48 h. (*e*) The vessel length was quantified from control and CSE-treated groups (*n* = 18). Data presented are the mean ± s.e.m. Unpaired Student's *t*-test, ^#^*p* < 0.0001, ***p < 0.001, **p < 0.01, *p < 0.05.

To interrogate the effects of CSE on angiogenesis, we firstly examined the viability of HUVECs treated with various concentrations of CSE. A CCK8 assay showed that the cellular viability was decreased in a dose-dependent manner (figure 1*a*). Two concentrations (50 µg ml^−1^ and 100 µg ml^−1^) were used in the following *in vitro* studies. We then performed tube formation assays to test the effects of CSE on the angiogenic capacities of HUVECs. The branch points in tube formation were quantified and the results showed a reduction in the number of branch points by CSE treatment. In addition, the endothelial network appeared to be disrupted after 8 h of CSE treatment (figure 1*b,c*). Furthermore, we investigated the alterations in vessel formation induced by CSE using *flk*::GFP transgenic zebrafish to determine the effects of CSE *in vivo*. The embryos were treated with CSE after 24 h post fertilization (hpf), at which time the dorsal aorta and the cardinal vein have developed and mostly formed [25]. The treated group showed a disorganized vasculature with a shorter length of blood vessels after 48 h exposure to CSE, suggesting that CSE induces disruption of the vascular network in zebrafish (figure 1*d*,*e*).

### 2.2. NRF2 was stimulated in cigarette smoke extract-induced vascular damage

**Figure 2.**
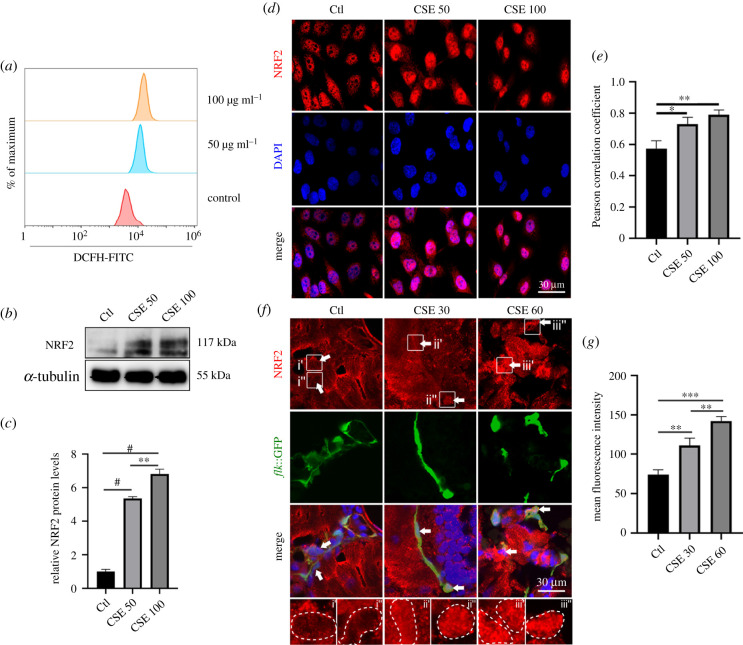
NRF2 was stimulated in CSE-induced vascular damage. (*a*) FACS for ROS generation in HUVECs exposed to CSE for 6 h. (*b*,*c*) NRF2 protein expression levels in HUVECs after 24 h of being exposed to CSE. (*d*,*e*) Representative immunofluorescence images and Pearson correlation coefficient analysis show nuclear localization of NRF2 and 4′,6-diamidino-2-phenylindole (DAPI) in HUVECs exposed to CSE for 24 h. (*f*,*g*) Representative immunofluorescence images and the statistics of average fluorescence intensity for NRF2 nuclear localization in vascular endothelial cells of *flk*::GFP zebrafish embryos exposed to CSE for 48 h (the arrows indicate the nuclei of the vascular endothelial cells) (*n* = 10). Data presented are the mean ± s.e.m. Unpaired Student's *t*-test, ^#^*p* < 0.0001, ***p < 0.001, **p < 0.01, *p < 0.05. DCFH, dichlorofluorescein diacetate; FITC, fluorescein isothiocyanate.

Next, we investigated the mechanism of how CSE disrupts vasculature formation. Given that CSE caused oxidative stress [26], we questioned whether the CSE-induced oxidative stress influenced vessel disorganization. We firstly confirmed the production of the oxidative stress response in HUVECs incubated with CSE for 6 h, marked by intracellular ROS accumulation and NRF2 activation. The cells were assayed by fluorescence activated cell sorting (FACS) analysis using dichlorofluorescin diacetate (DCF-DA) to examine the intracellular oxidative levels. The results revealed an increase in ROS production with CSE administration (figure 2*a*; electronic supplementary material, figure 1*a*). In addition, the protein levels of NRF2 were increased and NRF2 was translocated into the nucleus in CSE-treated HUVECs (figure 2*b–e*). Moreover, the expression of several key antioxidant factors including *NRF2*, *HMOX1* and *NOS2* was significantly upregulated during CSE induction (electronic supplementary material, figure 1*b*). Consistent with the observation in HUVECs, NRF2 signalling was enhanced in the nucleus of green fluorescent protein (GFP)-labelled *flk*^+^ cells (figure 2*f*,*g*). These master antioxidant regulators were dramatically induced in CSE-exposed zebrafish embryos (electronic supplementary material, figure 1*c*), confirming that CSE could induce NRF2 activation in disrupted vascular cells.

### 2.3. Reactive oxygen species clearance inhibited NRF2 activation to repair vasculature defects

**Figure 3.**
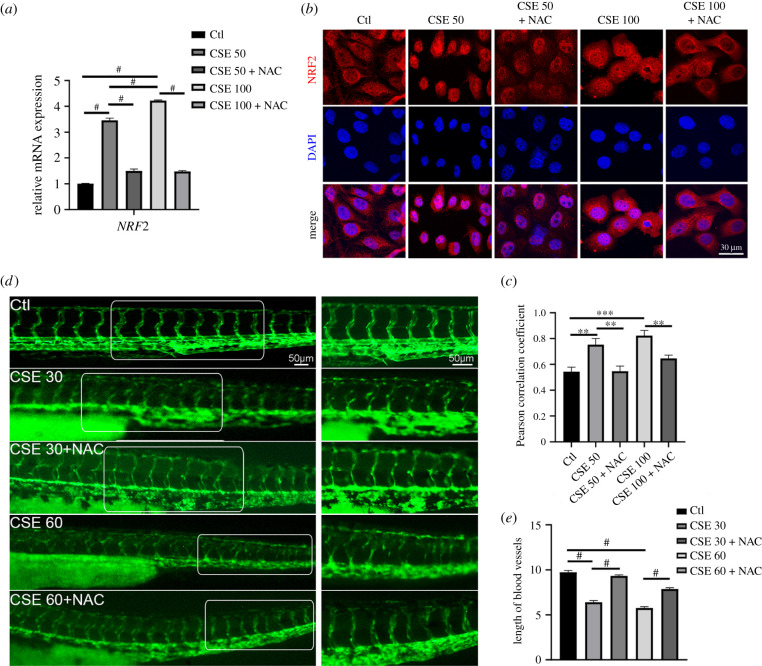
Elimination of ROS accumulation rescued the disrupted blood vessel. (*a*) The *NRF2* mRNA expression level was examined in HUVECs exposed to different CSE concentrations with or without NAC (1 mM) for 24 h. (*b*) Representative images indicated NRF2 nuclear localization from different treated groups. (*c*) Pearson correlation coefficient for the co-localization of NRF2 and DAPI. (*d*) Representative images for the *flk*::GFP zebrafish with or without NAC (5 mM) under different concentrations of CSE. (*e*) Blood vessel lengths were quantified for different treatments (*n* = 16). Data presented are the mean ± s.e.m. Unpaired Student's *t*-test, ^#^*p* < 0.0001, ***p < 0.001, **p < 0.01, *p < 0.05.

Since ROS levels were upregulated in HUVECs and zebrafish, we next investigated whether the inhibition of ROS accumulation restored the disruption of the vascular network. The *N*-acetyl cysteine (NAC) antioxidant neutralizing the effects of ROS was applied in CSE-treated HUVECs or zebrafish, and we found that NAC restored cell viability and angiogenic capacity interrupted by CSE exposure (electronic supplementary material, figure 2*a*–*c*). The addition of NAC in HUVECs dramatically decreased the levels of ROS upregulated by CSE and suppressed NRF2 transcriptional activation as well as nuclear translocation (figure 3*a*–*c*; electronic supplementary material, figure 2*d*), indicating that the vasculature disturbed by CSE could be recovered with NAC administration. Consistently, CSE only showed defects in the length of blood vessels in *flk*::GFP zebrafish, while the combination treatment with CSE and NAC repaired the vascular organization (figure 3*d*,*e*). Immunofluorescence staining was carried out, and the results revealed that NAC alleviated the transcriptional activation of NRF2 (electronic supplementary material, figure 2*e*,*f*). Therefore, angiogenic derangement caused by CSE was mainly due to aberrant oxidative stress and could be mitigated through the inhibition of ROS accumulation.

### 2.4. The fibronectin assembly was disrupted by oxidative stress in angiogenesis

**Figure 4.**
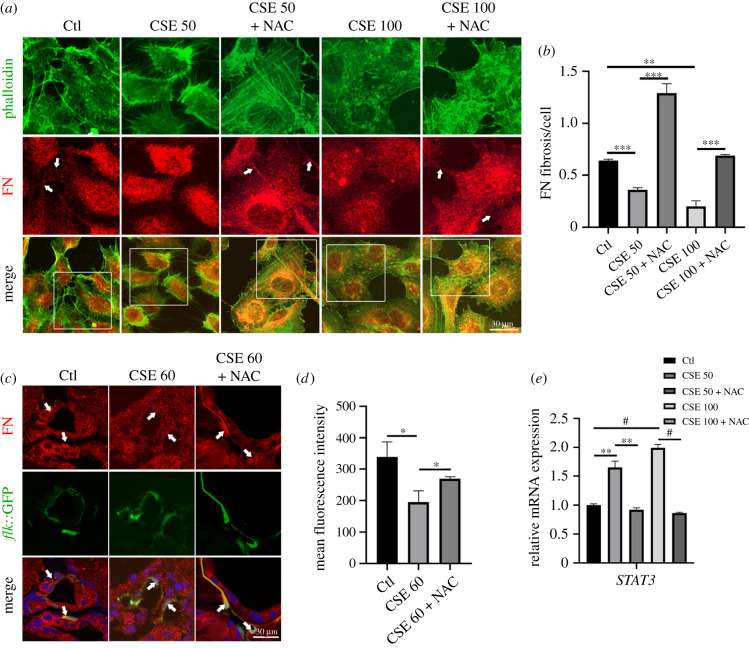
Oxidative stress induced by CSE caused abnormal FN assembly. (*a*) Immunofluorescence staining for FN and phalloidin in CSE-exposed HUVECs treated with or without NAC (the arrows indicate FN fibrils). (*b*) Quantification of the counts of FN fibrils in HUVECs. (*c*) Immunostaining for FN matrix among vascular endothelial cells in CSE-exposed zebrafish embryos treated with or without NAC (5 mM) for 48 h (the arrows indicate FN fibrils among the *flk*^+^ cells). (*d*) Quantitative analysis of the FN matrix among vascular endothelial cells (*n* = 9). (*e*) Quantitative polymerase chain reaction (qPCR) for the expression levels of *STAT3* mRNA in CSE-exposed HUVECs treated with or without NAC (1 mM) for 24 h. Data presented are the mean ± s.e.m. Unpaired Student's *t*-test, ^#^*p* < 0.0001, ***p < 0.001, **p < 0.01, *p < 0.05.

As FN assembly is one of the pivotal processes during vascular organization [27], we evaluated whether CSE induced abnormalities in FN assembly in HUVECs. Immunofluorescence staining showed that the extent of FN fibril formation was reduced and that actin filaments were disrupted in CSE-treated HUVECs (electronic supplementary material, figure 3*a*,*b*). In addition, FN assembly-associated genes were significantly upregulated (electronic supplementary material, figure 3*c*). To determine whether the deregulation of FN assembly by CSE is through ROS accumulation, NAC was applied in both HUVECs and zebrafish treated with CSE. FN fibrils and actin filaments were assembled in an organized manner when HUVECs were treated with CSE and NAC, while the assembly was damaged in the CSE-exposed group (figure 4*a*,*b*). Moreover, CSE exposure disturbed the continuous FN fibrils and induced unusual foci among the vascular endothelial cells of *flk*::GFP zebrafish (electronic supplementary material, figure 3*d*,*e*). These assembly defects could be partially rescued by NAC addition in embryonic zebrafish (figure 4*c*,*d*), which was consistent with our observation in CSE-treated HUVECs. These results suggested that oxidative stress induced by CSE disrupts FN assembly in vasculature formation.

### 2.5. NRF2 inhibition restored fibronectin assembly and vascular formation

**Figure 5.**
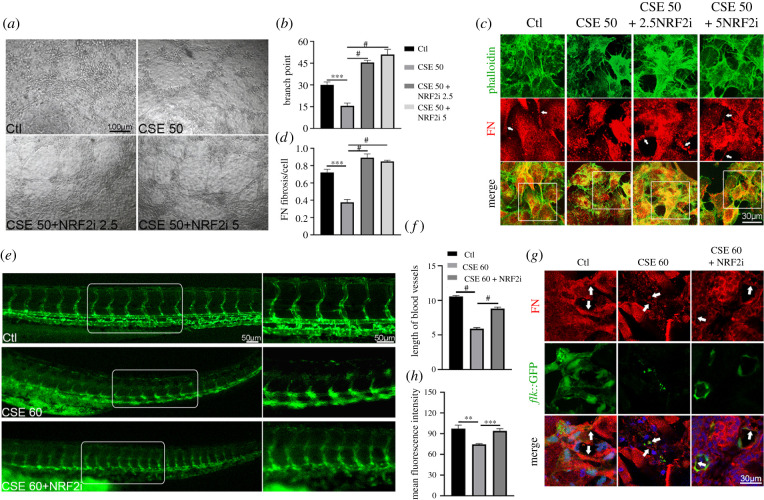
NRF2 inhibition restored abnormal angiogenesis induced by CSE. (*a*) Representative images showed angiogenic capacities in CSE-exposed HUVECs treated with or without NRF2 inhibitor (2.5/5 µM). (*b*) Quantification of the blood vessel branch points formed by HUVECs. (*c*) Representative immunofluorescence images of FN fibrils and phalloidin in HUVECs with CSE and NRF2 inhibitor treatment (the arrows indicate FN fibrils). (*d*) Quantification of the counts of FN fibrils in HUVECs. (*e*,*f*) *flk*::GFP zebrafish embryos were exposed to CSE with or without NRF2 inhibitor (25 µM) and vasculature formation was observed at 48 h; the blood vessel length was also quantified (*n* = 15). (*g*) Immunostaining for FN matrix among vascular endothelial cells in CSE-exposed *flk*::GFP zebrafish embryos with or without NRF2 inhibitor (the arrows indicate FN fibrils among the *flk*^+^ cells). (*h*) Quantification of the mean fluorescence intensity of FN among GFP-positive cells (*n* = 9). Data presented are the mean ± s.e.m. Unpaired Student's *t*-test, ^#^*p* < 0.0001, ***p < 0.001, **p < 0.01.

To determine whether NRF2 played critical roles in FN assembly and vasculature formation, HUVECs were treated with CSE in the presence of the potent NRF2 inhibitor ML385. We found that ML385 restored the tube network in a dose-dependent manner (figure 5*a*,*b*). FN fibrils were also more continuous in CSE-treated HUVECs with NRF2 inhibition (figure 5*c*,*d*). Similarly, the disrupted vascular formation was dramatically rescued in CSE-treated zebrafish embryos when exposed to ML385 (figure 5*e*,*f*). Concomitantly, ML385 reversed disorganization of the FN assembly induced by CSE in the vascular endothelial cells of zebrafish embryos (figure 5*g*,*h*).

### 2.6. NRF2 transcriptionally regulated STAT3 expression

**Figure 6.**
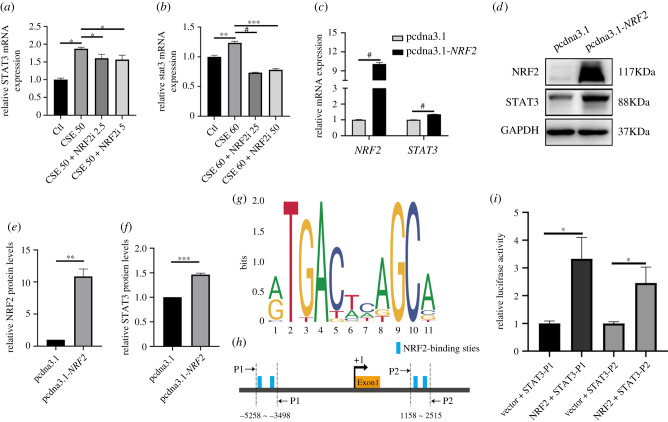
*NRF2* regulated *STAT3* at the transcriptional level. (*a*) qPCR for the expression levels of *STAT3* mRNA in CSE-exposed HUVECs treated with or without NRF2 (2.5/5 µM) inhibitor for 24 h. (*b*) qPCR for the expression levels of *stat3* mRNA in CSE-exposed zebrafish treated with or without NRF2 (25/50 µM) inhibitor for 24 h. (*c*) STAT3 mRNA expressions were examined with *NRF2* overexpression in 293FT cells at 48 h. (*d*–*f*) Analysis of the STAT3 protein level by immunoblot with *NRF2* overexpression in 293FT cells at 48 h. (*g*) NRF2-binding motifs from the JASPAR database. (*h*) Schematic representations of the *STAT3* promoter region. Two pairs of primers were designed to flank the potential NRF2-binding sites within the *STAT3* promoter. (*i*) Dual-luciferase assays of transcription with or without *NRF2* overexpression. Data presented are the mean ± s.e.m. Unpaired Student's *t*-test, ^#^*p* < 0.0001, ***p < 0.001, **p < 0.01, *p < 0.05.

We have demonstrated that NRF2 activation suppresses FN assembly. Next, we determined whether NRF2 acted on STAT3, the FN assembly-associated gene [24], to impact fibril formation. The expression of STAT3 at mRNA levels and protein levels was significantly upregulated in HUVECs or zebrafish embryos with CSE exposure (electronic supplementary material, figure 3*c*,*f*,*g*). The induction of *STAT3* expression at mRNA levels, as well as the NRF2 target gene *HMOX1*, was blocked by NRF2 inhibition in both CSE-treated HUVECs and zebrafish embryos (figure 6*a*,*b*; electronic supplementary material, figure 4*a*,*b*). Consistently, NRF2 overexpression led to an increase in STAT3 expression at both the mRNA and protein levels (figure 6*c*–*f*). To address the question of whether NRF2 transcriptionally regulated *STAT3* expression, the activities of the reporter containing the upstream regulatory region (URR) of *STAT3* were measured by the dual-luciferase reporter assay in NRF2-expressed cells. The URR includes four consensus NRF2-binding motifs [28,29]. The result showed the higher luciferase activities in the NRF2 overexpressed group, compared with the control (figure 6*g*–*i*).

In addition, the expression of *STAT3* transcripts was upregulated by CSE treatment, which was hindered by the NAC treatment down to an approximately normal level (figure 4*e*). We have previously shown that CSE induces NRF2 activation and that NAC suppresses NRF2 nuclear translocation as well as transcriptional activation (figure 3*a*–*c*; electronic supplementary material, figure 2*d*–*f*), which also supports our finding that NRF2 regulates *STAT3* transcription.

### 2.7. STAT3 inhibition rescued the assembly of fibronectin and angiogenesis

**Figure 7.**
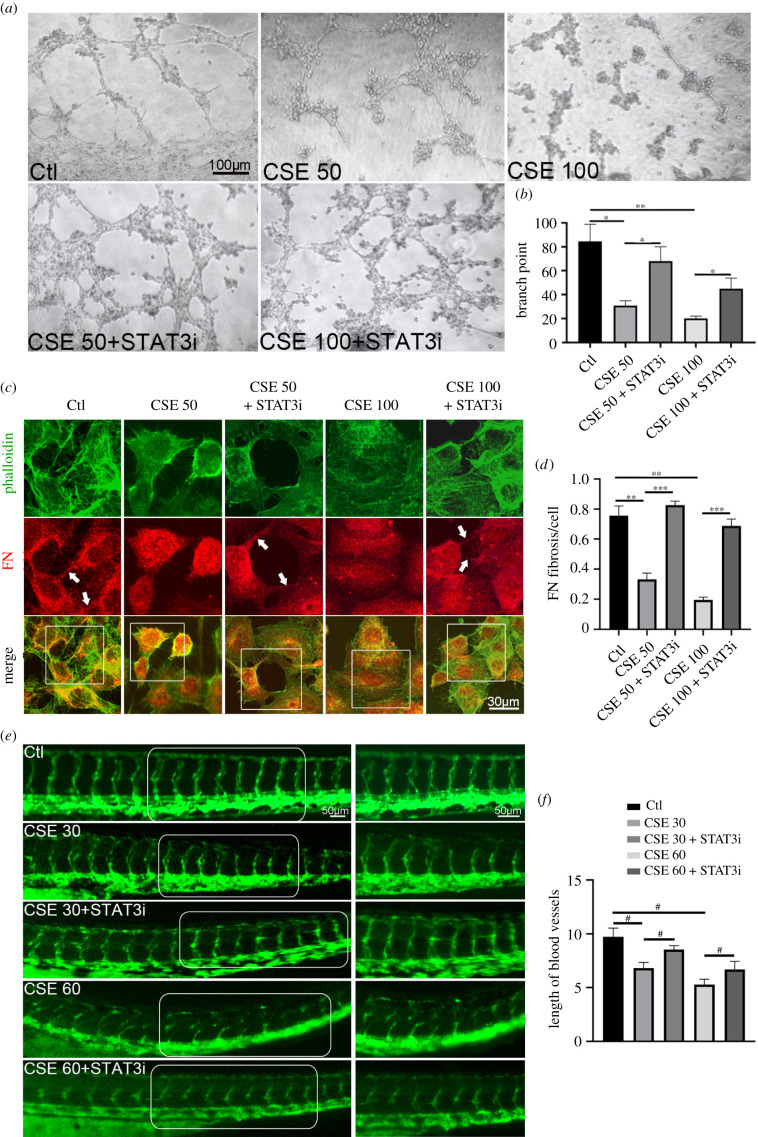
STAT3i rescued disrupted angiogenesis induced by CSE. (*a*) Representative images show angiogenic capacities in CSE-exposed HUVECs treated with or without STAT3i (5 µM) at 24 h. (*b*) Quantification of the blood vessel branch points formed by HUVECs. (*c*) Representative immunofluorescence images of FN and phalloidin expression in HUVECs with CSE treatment (the arrows indicate FN fibrils). (*d*) Quantification of the counts of FN fibrils in HUVECs. (*e*,*f*) *flk*::GFP zebrafish embryos were exposed to CSE with or without STAT3i (50 µM) and vasculature formation was observed at 48 h; the blood vessel length was also quantified (*n* = 20). Data presented are the mean ± s.e.m. Unpaired Student's *t*-test, ^#^*p* < 0.0001, **p < 0.01, *p < 0.05.

Our results have indicated that NRF2 regulates FN assembly and angiogenesis, and that NRF2 transcriptionally mediates STAT3 expression. We next investigated whether STAT3 impacted CSE-induced defects of FN assembly and angiogenesis, similar to what NRF2 does. The tube formation assay showed that the tube network was dramatically restored by the STAT3 inhibitor (STAT3i; S3I-201) (figure 7*a*,*b*). CSE-induced FN fibril reduction was also notably rescued with STAT3i administration (figure 7*c*,*d*). To further examine the effect of STAT3 on the failure of angiogenesis, STAT3i was applied to zebrafish embryos during CSE exposure, and the disrupted angiogenesis was remarkably recovered with inhibitor supplementation (figure 7*e*,*f*). Moreover, STAT3i evidently reversed disorganization of FN assembly induced by CSE among the vascular endothelial cells in zebrafish embryos (electronic supplementary material, figure 4*c*,*d*), supporting the important role of STAT3 in oxidative stress-induced vascular dysfunction.

## 3. Discussion

Cigarette smoke causes endothelial cell dysfunction in various cardiovascular diseases. However, the mechanisms underlying these procedures remain poorly studied, especially the ones focusing on the pathogenic effects of NRF2. Previous studies have shown that NRF2 plays essential roles in endothelial antioxidative defence, detoxification and inflammatory responses, whereas others found that NRF2 triggers cardiovascular damage and dysfunction [12,13]. Here, we demonstrate that cigarette smoke disrupts vascular organization during angiogenesis, and that this defect can be explained by aberrant FN deposition mediated through NRF2-STAT3 signalling in vascular endothelial cells.

The dual roles of NRF2 complicate our understanding of the pathology of cardiovascular diseases. For example, early studies reported that NRF2 activation could relieve the symptom of patients with circulatory problems [9,30]. Protandim induces NRF2 nuclear localization to alleviate oxidative stress-induced human coronary artery endothelial cell damage [31]. Metformin protects the cerebral vasculature from smoke toxicity through the enhancement of NRF2 activity [32]. These studies strongly suggested that NRF2 counteracts the oxidative stress stimulated by cigarette smoke. However, our findings show different results. Indeed, we have revealed that CSE-induced oxidative stress triggers the activation of NRF2 and other antioxidant gene expression. This raises the question of how NRF2 promotes defects of angiogenesis.

In our current studies, cigarette smoke induces ROS accumulation, NRF2 activation and STAT3 expression, as well as the consequent disruption of angiogenesis in HUVECs and endothelial cells of zebrafish. The dependence of the FN assembly and vessel formation on the former three factors is verified by their inhibitors accordingly. NAC, NRF2 inhibitor or STAT3i effectively ameliorate angiogenic damage and reduce related gene expression *in vitro* and *in vivo*. Collectively, these results demonstrate that cigarette smoke induces NRF2 activation by enhanced oxidative stress, leading to abnormal vasculature formation. Consistent with our findings, others reported that NRF2-mediated cardiac damage depends on redox imbalance [33–35].

We focused on FN as the downstream mediator of NRF2 activation to explain the disruption of angiogenesis induced by CSE. FN matrix assembly is fundamental for angiogenesis [18,36]. Generally, FN dimers bind with integrins to form the assembled complex, recruiting and connecting to the intracellular actin cytoskeleton, which in turn causes FN conformational changes, integrin clustering and FN dimer gathering [22]. FN expression can be regulated by NRF2 in a tissue-specific manner [16,17], but whether the assembly of FN depends on NRF2 is unknown. Our previous work suggested that stat3-efemp2a signalling is indispensable for the accuracy of FN assembly [24]. Here, our work determined that NRF2 activation disrupted FN assembly through STAT3. Consistently, we found that NRF2 regulated STAT3 transcript expression. Inhibition of NRF2 or STAT3 by inhibitors rescued CSE-induced defects of FN assembly and vessel formation. In addition, we also noticed that integrin *β*1 expression increased upon CSE treatment, raising the additional question of whether NRF2 interferes with FN organization in an integrin-dependent manner, which will be addressed in a future investigation.

Taken together, our study revealed the adverse role of NRF2-STAT3 signalling in FN assembly during vascular construction, which provides novel insights into our understanding of how cigarette smoke induces vascular toxicity.

## 4. Material and methods

### 4.1. Cell culture

HUVECs (obtained from ZTRI) were cultured in endothelial cell medium (Sciencell, Carlsbad, CA, USA). Human embryonic kidney cells (293FT) grown in Dulbecco's modified Eagle's medium (Gibco, Grand Island, NY, USA) containing 10% fetal bovine serum (FBS; Gibco, Grand Island, NY, USA). After reaching 80–90% confluence, cells were passaged by digesting with trypsin (Gibco, Grand Island, NY, USA). All cells were assayed at passages 3–10 and were maintained at 37°C in 5% CO_2_.

### 4.2. Zebrafish strain

Zebrafish were raised and maintained by the following standard procedure. The transgenic line (Tg (*flk*::GFP)) was a gift from Prof. Mo's lab. All experiments involving the use of animals were conducted in compliance with approved guidelines. The animal protocols were approved by the Ethics Committee of Chongqing Medical University.

### 4.3. Preparation of cigarette smoke extract solutions

CSE was prepared as previously described [37]. Briefly, Kentucky reference cigarettes 3R4F from the University of Kentucky (Lexington, KY, USA) were conditioned at 22 ± 1°C and 60 ± 3% relative humidity for 48 h before testing. According to the ISO standard smoking regimen (ISO 4387:2000), 20 cigarettes were smoked with a Borgwaldt-KC RM20H smoking machine (Borgwaldt KC, Hamburg, Germany). Mainstream cigarette smoke was passed through a 92 mm Cambridge filter (Whatman, Little Marlow, UK) and was dissolved with dimethyl sulfoxide (Sigma-Aldrich, St Louis, MO, USA) as 10 mg ml^−1^ of CSE stock solution by shaking for 30 min at room temperature. The CSE solution was filtered with a 0.22 µm filter and was stored at −80°C. All treated CSE solutions contained the same concentration of DMSO for this study.

### 4.4. Cell viability assay

A Cell Counting Kit-8 (Dojindo, Japan) was used to assay cell viability. HUVECs were seeded into a 96-well plate in 100 µl of cell suspension (5000 cells/well) and incubated overnight. Subsequently, cells were treated with CSE solutions at relative concentration gradients with/without NAC (1 mM) for 24 h. Then, cells were rinsed with phosphate-buffered saline (PBS) twice and 10 µl of CCK-8 reagent was added into each well containing 100 µl of medium. After incubating for 1 h, the optical density value was measured at 450 nm with a microplate reader (Thermo Scientific^TM^, Waltham, MA, USA).

### 4.5. Zebrafish treatment and vascular assay

Fertilized eggs were torn from the yolk membrane 1 day after spawning. All the treatments in zebrafish embryos were performed at 24 hpf. The blood vessels (GFP) were observed with a stereographic fluorescence microscope (Carl Zeiss, Göttingen, Germany) after 48 h of treatment with CSE with/without NAC or ML385 or S3I-201 (Topscience, Shanghai, China). The length of the blood vessels was measured by measuring software (ImageJ 1.8.0_172).

### 4.6. Tube formation assay

Cool Matrigel (Corning, MA, USA) was laid on the bottom of the 96-well plate. HUVECs starved under the ECM containing 0.2% FBS overnight were seeded into a 96-well plate at 100 µl of cell suspension (1 × 10^4^ cells/well) with/without CSE and NAC/ML385/S3I-201 for 8–10 h. The vascular structures of the HUVECs were imaged by inverted microscope imaging.

### 4.7. Gene expression assay

Total RNA was extracted from whole dish cells or zebrafish embryos (approximately 10 embryos per group) using RNAiso Plus reagent (Takara, Japan). cDNA was generated using Hiscript II qRT SuperMix II (Vazyme Bio, Nanjing, China). qPCR was performed using ChamQ Universal SYBR qPCR Master Mix (Vazyme Bio, Nanjing, China) on a QuantStudio 1 (Thermo Scientific^TM^, Waltham, MA, USA). The fold change of target genes was calculated with the 2^−ΔΔCt^ method.

### 4.8. Western blot assay

Cells were lysed using radioimmunoprecipitation assay (RIPA) buffer, and harvested proteins were separated by electrophoresed sodium dodecyl sulfate–polyacrylamide gel electrophoresis (SDS-PAGE), followed by electrophoretic transfer onto a polyvinylidene fluoride membrane (Merck Millipore, MA, USA). The membranes were blocked with 5% skimmed milk in Tris-buffered saline containing 0.05% Tween-20 (TBST) for 1 h at room temperature. After incubation with primary and secondary antibodies, ECL blotting reagents (Vazyme Bio, Nanjing, China) were used for immunoblotting detection, and proteins were quantified using ImageJ software. The primary antibodies were purchased from Proteintech (Wuhan, China): anti-NRF2 (16396-1-AP), anti-FIBRONECTIN (15613-1-AP), anti-STAT3 (10253-2-AP), anti-α-TUBULIN (66031-1-Ig) and anti-GAPDH (60004-1-Ig).

### 4.9. Luciferase reporter assay

According to a literature review, there were four potential NRF2-binding sites in the –5000 bp to 2000 bp region of the transcription start site of STAT3. Two sequences containing two of these binding sites named STAT3-P1; STAT3-P2 were amplified by PCR and cloned into a PGL3-promoter vector. The coding sequence (CDS) region of human NRF2 was cloned into the pcDNA3.1 vector. A Dual Luciferase Reporter Assay Kit (Beyotime, Beijing, China) was used for luciferase assays. Briefly, 293FT cells were seeded at 8 × 10^4^ cells per well in a 24-well plate, co-transfected with 200 ng of luciferase reporter construct, 40 ng of pRL-TK plasmid and using 720 ng polyethylenimine (PEI) reagent per well. Cells were lysed in lysis buffer after 48 h transfection. Luminescence was assayed by a plate reader (Thermo Scientific^TM^, Waltham, MA, USA). Three biological replicates were used in each condition. Firefly luciferase signal was normalized to Renilla luciferase signal to account for transfection efficiency.

### 4.10. Immunofluorescence staining

Immunofluorescence staining was performed as previously described [23]. Briefly, treated cells grown on glass coverslips were washed three times with PBS and fixed in 4% paraformaldehyde (PFA) for 10 min at room temperature. Zebrafish embryos were fixed overnight at 4°C in 4% PFA in PBS and were cut into 10 µm sections. For staining, cells or sections were permeabilized with 0.3% Triton X-100 in PBS containing 5% bovine serum albumin (BSA; Sigma-Aldrich, St Louis, MO, USA) for 30 min. After primary antibodies were incubated overnight at 4°C, samples were washed with PBS and incubated with fluorescein-conjugated antibodies or phalloidin (Beyotime, Beijing, China) for 1 h at room temperature. All immunolabelled samples were viewed and photographed using confocal microscopy (Nikon, ECLIPSE Ti).

### 4.11. Flow cytometry for determination of reactive oxygen species

ROS generation was analysed by the fluorescent probe DCF-DA (Sigma-Aldrich, St Louis, MO, USA). HUVECs were plated into a six-well plate (1.5 × 10^5^ cells/well) and cultured overnight. The cells were treated with different concentrations of CSE with/without NAC for 6 h, then 10 µM DCF-DA was added and the cells were incubated for 30 min at 37°C in the dark. Cells were harvested and were measured by flow cytometer (Beckman Coulter, CA, USA). The results were analysed by FlowJo v. 10 software.

### 4.12. Statistics

All statistical analyses were performed with the unpaired Student's *t*-test. *p*-value ≤ 0.05 was considered statistically significant. All statistical data were derived from at least three independent experiments.

## Data Availability

The data are provided in the electronic supplementary material [38].
